# Network toxicology reveals key genes of amiodarone induced pulmonary fibrosis: based on machine learning and SHAP analysis

**DOI:** 10.3389/fphar.2026.1755004

**Published:** 2026-02-23

**Authors:** Xiaoyan Yan, Ying Liu, Rui Fan

**Affiliations:** 1 Department of Geriatric Medicine, Affiliated Hospital of Shandong University of Traditional Chinese Medicine, Jinan, Shandong, China; 2 Department of Pulmonary Medicine, Xiangyang Hospital affiliated to Hubei University of Chinese Medicine, Xiangyang, Hubei, China; 3 Respiratory and Critical Care Medicine Department, Provincial Hospital Affiliated to Shandong First Medical University, Jinan, Shandong, China

**Keywords:** ADORA3, AGER, amiodarone-induced pulmonary fibrosis, CTSK, FLT3, machine learning, SHAP analysis

## Abstract

**Background:**

Amiodarone (AMD), a highly effective Class III antiarrhythmic drug, has its clinical utility limited by the risk of inducing a serious adverse effect, amiodarone-induced pulmonary fibrosis (AIPF). The pathogenesis of AIPF remains poorly elucidated, particularly the hub driver genes, which hinders early diagnosis and targeted intervention.

**Methods:**

This study employed an integrative approach combining network toxicology, machine learning (ML), and *in vitro* validation to identify hub genes in AIPF. Potential AMD targets and pulmonary fibrosis (PF)-related genes were obtained from toxicity databases and transcriptomic data (GEO datasets), respectively, and intersected to identify candidate AIPF targets. Multiple ML models were constructed, and SHAP (Shapley Additive exPlanations) analysis was used to interpret the model and rank feature importance. Molecular docking and dynamics simulations assessed the binding of AMD to the core targets. Key findings were experimentally validated in an AMD-induced human bronchial epithelial (BEAS-2B) cell model using qRT-PCR, Western blot, and functional assays.

**Results:**

Bioinformatics analysis identified eight candidate hub genes for AIPF. The glmBoost + GBM model demonstrated superior predictive performance (AUC = 0.845). SHAP interpretability analysis identified Cathepsin K (CTSK), Adenosine A3 Receptor (ADORA3), and Advanced Glycosylation End Product-Specific Receptor (AGER) as the most important predictors. Molecular simulations confirmed stable binding between AMD and these target proteins. *In vitro* experiments showed that AMD treatment significantly upregulated CTSK and downregulated ADORA3 and AGER at both mRNA and protein levels in BEAS-2B cells, and enhanced cell migration and invasion.

**Conclusion:**

This study identifies CTSK, ADORA3, and AGER as key genes in AIPF pathogenesis through a comprehensive bioinformatics and ML approach. Their dysregulation in lung epithelial cells likely promotes fibrosis through modulating extracellular matrix metabolism, inflammation, and cell motility. These findings provide novel insights into AIPF mechanisms and highlight potential biomarkers and therapeutic targets.

## 1 Introduction

Amiodarone (AMD) is a Class III antiarrhythmic agent being utilized in clinical practice. Its main pharmacological effect is to significantly prolong the action potential duration and effective refractory period of cardiomyocytes, thereby inhibiting the triggering and maintenance of rapid arrhythmias (Mason, 1987; Freedman and Somberg, 1991). However, its therapeutic utilization is constrained by a range of severe non-cardiac adverse effects, with AMD-induced pulmonary toxicity (AIPT) being the most concerning and potentially lethal (Retz and Martin, 1992; Okayasu et al., 2006). AIPT manifests as a spectrum of interstitial lung diseases, with amiodarone-induced pulmonary fibrosis (AIPF) representing the most severe and irreversible form (Retz and Martin, 1992). Clinically, AIPF has an insidious onset, with early manifestations often including dry cough, dyspnea, fatigue, and weight loss. Chest CT scans can reveal diffuse infiltration, ground-glass opacities, or thickened reticular structures in both lungs. AIPF progresses rapidly, often leading to severe respiratory failure (Hamilton et al., 2020; Feduska et al., 2021). This poses significant challenges for clinical management, as diagnosis typically requires ruling out other PF (Papiris et al., 2010).

The mechanism of PF involves repeated injury to alveolar epithelial cells triggering epithelial-mesenchymal transition (EMT), alongside endothelial cells undergoing endothelial-mesenchymal transition (EndMT) to transform into mesenchymal cells. This subsequently activates fibroblast proliferation and myofibroblast differentiation, leading to excessive extracellular matrix deposition and pathological fibrosis (Weinstein et al., 2020; Moss et al., 2022). However, the key genes and pathogenesis of AIPF remain unelucidated to date. AIPF is marked by an cytotoxic injury and a dysregulated inflammatory response, leading to abnormal collagen deposition and remodeling of the extracellular matrix (ECM), ultimately resulting in irreversible PF (Reinhart et al., 1996). Due to its long half-life and tissue accumulation properties, combined with its metabolism in the liver to the toxic metabolite desethylamiodarone (Bognar et al., 2017), AMD may cause severe pulmonary toxicity, manifesting as acute lung injury or chronic PF. Desethylamiodarone can lead to phospholipidosis, oxidative stress, and activation of pro-inflammatory and pro-fibrotic pathways (Feduska et al., 2021). Despite these findings, the precise molecular pathways by which AIPF induces persistent fibroblast proliferation and ECM formation following alveolar epithelial cell injury remain inadequately characterized (Quyyumi et al., 1983). Identifying hub genes driving AIPF progression is crucial for determining early diagnostic biomarkers and developing personalized treatments.

Traditional methods for identifying key AIPF genes typically rely on small-scale expression analyses, which inherently limit the scope of the study and may overlook the impact of gene contribution. In recent years, advances in molecular biology and bioinformatics have provided powerful tools for identifying key AIPF genes. Machine learning (ML) can assimilate and scrutinize intricate multigenomic data, offering a robust method for pinpointing essential genes implicated in the pathogenesis and advancement of AIPF. Moreover, interpretative instruments like SHAP (Shapley Additive exPlanations) may clarify the operational process and the contribution of each feature variable in a ML model, thereby seamlessly integrating prediction accuracy with biological interpretability.

This study aims to identify hub genes in AIPF using various bioinformatics methods and validate them *in vitro*. We acquired GEO transcriptome data associated with AMD exposure and PF pathogenesis, constructed ML-based predictive models, and validated them on independent GEO datasets. Subsequently, we employed SHAP analysis to rank the importance of hub genes, further clarifying their contributions to AIPF model predictions. The results will provide new insights into the hub genes and potential mechanisms of AIPF and lay the foundation for future clinical interventions.

## 2 Methods

### 2.1 Acquisition of chemical components and targets of amiodarone

We used computer simulation analysis to identify AMD-related genes. First, we identified the target organs for amiodarone toxicity using the Protox database (https://tox.charite.de/protox3/) (Banerjee et al., 2018; Banerjee et al., 2024). Then, We obtained its canonical molecular structure and SMILE formula from the PubChem database (https://pubchem.ncbi.nlm.nih.gov/) (Kim et al., 2024). To identify potential therapeutic targets for AMD, we performed computational target prediction for the active compounds. This process involved querying each compound against three independent databases: ChEMBL (https://www.ebi.ac.uk/chembl/) (Davies et al., 2015; Zdrazil et al., 2024), Similarity Ensemble Approach (SEA) (https://sea.bkslab.org/) (Keiser et al., 2007), and SwissTargetPrediction platform (https://www.swisstargetprediction.ch/) (Daina et al., 2019; Daina and Zoete, 2024). After deduplication, a total of 280 high-confidence AMD-related targets were obtained.

### 2.2 Acquisition of disease-related targets

We obtained five PF gene expression datasets (GSE17978, GSE24206, GSE32537, GSE40839, and GSE47460) from the GEO database to explore PF-related genes (Edgar et al., 2002; Barrett et al., 2013). All datasets were retrieved from the GEO database in MINiML format, which contained comprehensive platform, sample, and series metadata. Raw data were normalized using the preprocess Core package. Probe IDs were converted to gene symbols according to corresponding platform annotation files. Batch effect correction was performed using the limma package for samples derived from different batches within the same dataset. When integrating data across multiple datasets or different platforms, only gene symbols common to all datasets were retained. Each dataset or platform was designated as a distinct batch, and batch effects were uniformly adjusted. The effectiveness of batch correction was evaluated by principal component analysis (PCA) conducted before and after normalization. The first four datasets were used as a training cohort. After batch effect correction and standardization, differential expression analysis was performed on this cohort to identify Differentially expressed genes (DEGs). Simultaneously, Weighted Gene Co-Expression Network Analysis (WGCNA) was applied to identify gene modules highly associated with the PF phenotype. The module with the strongest association (MEred) was selected. The union of DEG genes and WGCNA (MEred) genes yielded 233 PF-related genes. All data processing and statistical analysis were performed in the R 4.5.1 environment.

### 2.3 Identification of AIPF targets and functional enrichment analysis

To determine the core targets of AIPF, we intersected 280 AMD-related targets with 233 PF-related genes to derive putative AIPF hub genes. We imported the target gene list into the STRING database (version 11.5) to construct a protein-protein interaction (PPI) network, using *Homo sapiens* as the species and selecting a medium confidence threshold (>0.4). We exported TSV-formatted data containing nodes, edges, and interaction confidence scores for subsequent network visualization and topological analysis. We subsequently employed the clusterProfiler package in R 4.5.1 to conduct Gene Ontology (GO) and Kyoto Encyclopedia of Genes and Genomes (KEGG) pathway enrichment studies on these hub genes, considering entries with a corrected *p* < 0.05 as statistically significant.

### 2.4 Machine learning and SHAP analysis

To further explore and screen hub AIPF genes, we constructed a ML approach combined with SHAP analysis. 113 ML prediction models were constructed based on transcriptome data, and their performance was validated through independent cohorts using Receiver Operating Characteristic curve (ROC). To determine the optimal predictive model, we conducted a systematic evaluation and comparison based on the model predictive performance and its alignment with the target cohort’s demographic and clinical characteristics. Ultimately, the best algorithm was selected through stratified cross-validation and independent validation on a test set, ensuring the model possesses optimal generalization capability and clinical applicability. The best-performing prediction model was selected for further analysis. SHAP interpretability analysis was applied to quantify the contribution and direction of each gene′s characteristics in the model prediction based on its SHAP value, thereby identifying and ranking hub genes for AIPF classification.

### 2.5 Molecular docking analysis and molecular dynamics simulation

We performed molecular docking simulations to clarify the binding sites between AMD and its target proteins. We obtained the protein structure files of the AIPF hub genes from the PDB database and downloaded the 3D molecular file of AMD from PubChem. Molecular docking was performed using AutoDock Vina, and the data was converted to PDBQT format using MGLTools or OpenBabel. The center coordinates of the binding pocket and the search box size (x, y, z lengths) were defined in the receptor PDBQT, a configuration file (config.txt) was generated, and the exhaustiveness parameter was set. The docking results were evaluated using binding energy (ΔG, kcal/mol); a binding energy below −5.0 kcal/mol indicated strong binding activity. Finally, the binding sites and intermolecular interaction patterns were visualized and analyzed using PyMOL.

Molecular dynamics simulations were performed using the GROMACS 2022, the system was constructed using the AMBER99SB force field and placed in a periodic boundary box, followed by energy minimization to eliminate steric clashes. Subsequently, the system was thoroughly equilibrated successively under NVT and NPT ensembles, employing the V-rescale method for temperature coupling and the Parrinello-Rahman method for pressure coupling, until the temperature, density, and energy of the system stabilized. Following equilibration, a production simulation was conducted for a 50 ns with an integration time step of 2 fs. Short-range interactions were treated using a cutoff distance, while long-range electrostatic interactions were calculated using the Particle Mesh Ewald method. Hydrogen bonds were constrained using the LINCS algorithm. Trajectory data were saved at defined intervals for subsequent structural and dynamical analyses. We employed the built-in “g_sham” script and the “xpm2txt.py” script within GROMACS 2022 to calculate Gibbs free energy based on the RMSD and Rg values of stable complexes, and generated 2D Gibbs free energy contour plots.

### 2.6 Transwell migration/invasion assay and wound healing assay

We used AMD-induced BEAS-2B cell model for *in vitro* experiments. The CCK-8 assay was employed to evaluate the proliferation-inhibitory effect of AMD on BEAS-2B cells, thereby determining its optimal intervention concentration and providing a critical dose reference for subsequent mechanism studies. Cells were resuspended in serum-free medium. For the migration assay, cells were seeded at a density of 1 × 10^6^ cells/mL into the upper chamber of Transwell filters (8 μm pore size, Corning). The lower chamber was filled with medium containing 10% FBS to induce migration. For the invasion assay, matrix gel (50 μL/well, Servicebio) was pre-coated onto the lower chamber, and cells were seeded at a density of 5 × 10^5^ cells/mL. After 24–48 h at 37 °C, remove non-migrated cells from the upper chamber. Fix with 4% paraformaldehyde and stain with 0.1% crystal violet. Count cells in the lower chamber across 5 randomly selected fields. Migration/invasion rate = (experimental group cell count/control group cell count) × 100%. Seed cells (5 × 10^5^ cells/well) into 6-well plates. Upon 90% confluence, create linear scratches using sterile pipette tips. Wash debris with PBS and switch to 1% FBS medium to inhibit proliferation. Photograph the fixed sites at 0 h and 24 h. $\text{Healing rate}=\left(\text{Initial area}-24h\text{ area}\right)/\text{Initial area}\times 100\%$ .

### 2.7 AIPF hub genes and proteins expression in AMD-induced BEAS-2B cells

BEAS-2B cells were cultured in DMEM +10% FBS and treated with 10 μM AMD for 48 h to establish an epithelial AIPF model. Total RNA was extracted using TRIzol; concentration and purity were assessed by NanoDrop. One microgram of RNA was reverse-transcribed into cDNA. mRNA levels of CTSK, ADORA3, FLT3, and AGER were quantified by SYBR Green qPCR with gene-specific primers. Protein expression was evaluated by Western blot: proteins were extracted in RIPA buffer, quantified by BCA assay, separated by SDS-PAGE, transferred to PVDF membranes, and blocked with 5% non-fat milk. Membranes were incubated overnight at 4 °C with primary antibodies against CTSK, ADORA3, AGER, and FLT3, then with HRP-conjugated secondary antibodies. Subcellular localization and expression were assessed by immunofluorescence: cells on coverslips were fixed, permeabilized, blocked, incubated overnight at 4 °C with the same primary antibodies, then with fluorochrome-conjugated secondaries; nuclei were counterstained with DAPI. Confocal images were acquired and fluorescence intensity quantified in ImageJ. Relative mRNA and protein levels were calculated using the 2^−ΔΔCT^ method with GAPDH as the internal control.

BEAS-2B cells, STR correctly identified, were purchased from Wuhan Pronosai, catalog number: CL-0016, specification: 1 × 10^6^ cells/T25 culture flask, growth medium: Ham’s F-12K + 10%FBS+1%P/S. The normal human lung epithelial cells BEAS-2B used in this study were purchased from Shanghai Zhongqiao Xinzhou Biotechnology Co., Ltd., catalog number ZQ0381. Anti-Cathepsin K Rabbit pAb was purchased from Servicebio (product number: GB111276). Anti-Adenosine A3 Receptor/A3AR Rabbit pAb was purchased from Servicebio, accession number: GB112420; Anti-RAGE Rabbit pAb was purchased from Servicebio, product number: GB11278. Anti-FLT3 Rabbit pAb was purchased from Servicebio (product number: GB111358). Anti-GAPDH Rabbit pAb was purchased from Servicebio, accession number: GB11002. All primers sequences are listed as follows: GAPDH -F: 5′-CCT​GCA​CCA​CCA​ACT​GCT​TA-3′; GAPDH-R: 5′-GGC​CAT​CCA​CAG​TCT​TCT​GAG-3′; CTSK-F: 5′-CAG​CAG​ACG​CAG​ACA​TTA​CG-3′, CTSK-R: 5′-GAT​GCC​AGG​GTA​CAG​CTC​TC-3′; ADORA3-F: 5′-TGG​TCG​TCG​TCT​TCT​TCA​TC-3′, ADORA3-R: 5′-CAA​AGC​CAC​AAG​GAA​GAC​CT-3′; FLT3-F: 5′-TGC​AAG​CAA​GGA​CGT​GAA​G-3′, FLT3-R: 5′-GAC​TTG​CCA​GGA​AGG​TGA​TG-3′; AGER-F: 5′-GGA​AGG​AAC​GTC​TCA​ACC​AA-3′, AGER-R: 5′-GGT​CTG​GGC​ATT​TAT​CCT​CA-3′.

## 3 Statistical methods

GEO transcriptomic data underwent batch correction (limma package) followed by differential expression analysis using a two-tailed Welch’s t-test. DEGs were identified at a false discovery rate (FDR) < 0.05. ML model performance was evaluated by the AUC on both training and independent test sets. *In vitro* experimental data are presented as mean ± standard deviation. Intergroup comparisons were performed using Student’s t-test (two groups) or one-way analysis of variance (ANOVA). Post-hoc multiple comparisons were conducted using LSD-t/Dunnett-t tests based on Levin’s test results, with a significance level set at *p* < 0.05.

## 4 Result

### 4.1 Identification of AMD target proteins

**FIGURE 1:**
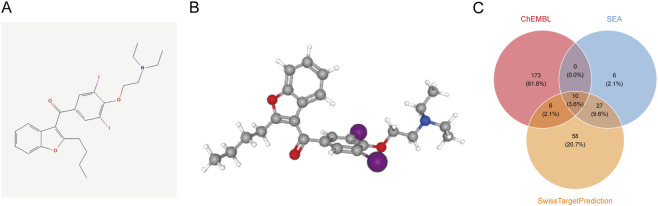
The Chemical Structure of AMD and Its Potential Protein Targets. **(A)** 2D planar chemical structure of AMD; **(B)** 3D minimum-energy conformation of AMD; **(C)** the Venn diagram of predicted human protein targets of AMD from three independent databases: ChEMBL, SEA, and SwissTargetPrediction.

This study first conducted computer simulation analysis to determine the target sites of AMD in the human body. The definitive molecular structure of AMD (SMILES: CCCCC1 = C(C2 = CC = CC = C2O1)C(=O)C3 = CC(=C(C(=C3)I)OCCN(CC)CC)I) was sourced from the PubChem, as shown in Figures 1A,B. By consulting the Protox database, we found that the predicted LD50 of AMD is 3,000 mg/kg, and the predicted Toxicity Class is 5/6 (1/6 is the most toxic). Table 1 presents the Toxicity Model Report, demonstrating that ADM exhibits the most significant pulmonary toxicity, mostly through immunotoxicity. Subsequently, parallel analysis was conducted utilizing three target prediction platforms: the ChEMBL, SEA, and the SwissTargetPrediction platform.

**TABLE 1:** AMD toxicity model report.

Classification	Target	Prediction	Probability
Toxicity end points	Immunotoxicity	Active	0.99
Organ toxicity	Respiratory toxicity	Active	0.96
Organ toxicity	Neurotoxicity	Active	0.8
Metabolism	Cytochrome CYP2D6	Active	0.75
Metabolism	Cytochrome CYP2C9	Active	0.70
Organ toxicity	Hepatotoxicity	Active	0.69
Toxicity end points	Ecotoxicity	Active	0.69
Molecular initiating events	Achetylcholinesterase (AChE)	Active	0.69

The prediction results from these three platforms are summarized in Figure 1C. The ChEMBL database identified 189 distinct targets, the SwissTargetPrediction identified 101, and the SEA technique identified 43. Remarkably, only 43 targets were identified in common by those three platforms, illustrating the complementarity of the various computational approaches. By consolidating all unique targets, a total of 280 putative high-confidence targets of AMD were identified, establishing the foundational dataset for further analysis.

### 4.2 Identification of PF-related genes

**FIGURE 2:**
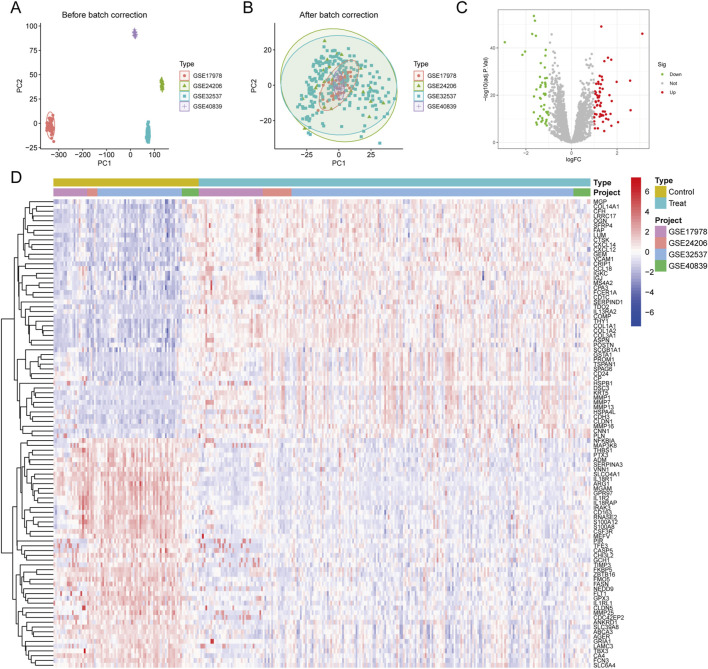
DEGs Analysis of PF. **(A,B)** Comparison of PCA plots before **(A)** and after **(B)** batch correction demonstrates the effective mitigation of batch-specific clustering. **(C)** Volcano plot of differentially expressed genes (DEGs). Genes are plotted based on the magnitude of log_2_ FC and statistical *p* value. Red dots represent significantly upregulated genes, green dots represent significantly downregulated genes, and gray dots indicate non-significant genes. **(D)** Heatmap displays gene expression levels, with red and blue representing high and low expression relative to the mean, respectively. Rows represent individual genes, while columns represent sample origins.

This study obtained five separate gene expression datasets from the GEO database (GSE17978, GSE24206, GSE32537, GSE40839, and GSE47460) to uncover hub genes linked to PF pathogenesis. The initial four datasets (GSE17978, GSE24206, GSE32537, and GSE40839) were amalgamated as training sets for preliminary gene screening, whereas GSE47460 was preserved as a subsequent validation set (Table 2). Batch correction was performed to mitigate the confounding effects of technical variation arising from different experimental runs, thereby ensuring that the downstream analyses more accurately reflect underlying biological differences. First, we normalized and corrected for batch effects in the training set to eliminate abiotic variation caused by differences in experimental platforms and techniques (Figures 2A,B). Subsequently, differential expression analysis was performed on the corrected data using R 4.5.1. A total of 109 DEGs were identified, including both upregulated and downregulated genes. The results of the differential analysis were visualized using a volcano plot (Figure 2C). A heat map (Figure 2D) was also created, showing the expression patterns of the 50 most significantly DEGs across each sample. The volcano plot and heat map visually demonstrate the differences in gene expression profiles between the Treat and Control groups. We can observe that in the Treat group, genes such as MGP, COL14A1, and CFH are highly expressed, while genes including NFKBIA, MAP3K8, and THBS1 are downregulated.

**TABLE 2:** GEO data.

GEO data	Platforms	Control samples	Experimental samples	Subject type
GSE17978	GPL570	20	38	IPF
GSE24206	GPL570	6	17	Early IPF/advanced IPF
GSE32537	GLP6244	50	167	IPF/NSIP/RB-ILD/DIP/UF
GSE40839	GLP96	10	11	Scleroderm-ILD/UIP
GSE47460	GLP6480	17	61	ILD
	GLP14550	91	193	ILD

**FIGURE 3:**
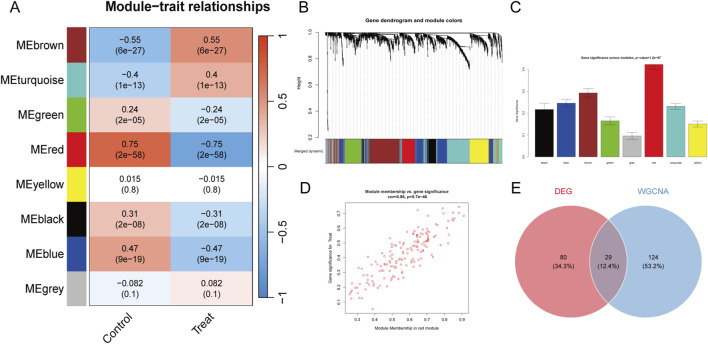
WCGNA Analysis of PF. **(A)** Module-trait relationship heatmap, showing the module signature genes and key traits identified by WGCNA analysis. Each row represents a module color, and each column represents a trait. Positive correlations are shown in red, and negative correlations are shown in blue. **(B)** Hierarchical clustering dendrogram of genes and identified co-expression modules. Genes were clustered based on a dissimilarity matrix (1-TOM). **(C)** Gene significance across modules. **(D)** Scatter plot showing the strong positive correlation (cor = 0.86, *p* = 5.7e-46) between module membership and gene significance for the treatment phenotype. **(E)** Venn diagram summarizing DEGs, WGCNA (MEred), and their union.

Then, we utilized WGCNA to find the gene clusters most closely connected with the PF phenotype. Through the construction of a scale-free co-expression network, we identified several gene modules, each denoted by a distinct color. The examination of module-trait relationships indicated that the red module (MEred) exhibited the strongest association with PF disease state (correlation coefficient = 0.75, *p* = 2 × 10^−58^), as illustrated in Figures 3A–C. Scatter plot depicting a strong positive correlation (cor = 0.86, p = 5.7 × 10^−46^) between module membership and gene significance for the treatment group, Each point represents a gene within the red module (Figure 3D). Consequently, the 153 genes within this module were designated as PF-related module genes and utilized for further investigation. We integrated the genes identified by the two methods described above and took the union of DEGs and WGCNA (MEred) genes. As shown in Figure 3E, we obtained 233 PF-related genes.

### 4.3 Identification of hub genes in AIPF and enrichment analysis

**FIGURE 4:**
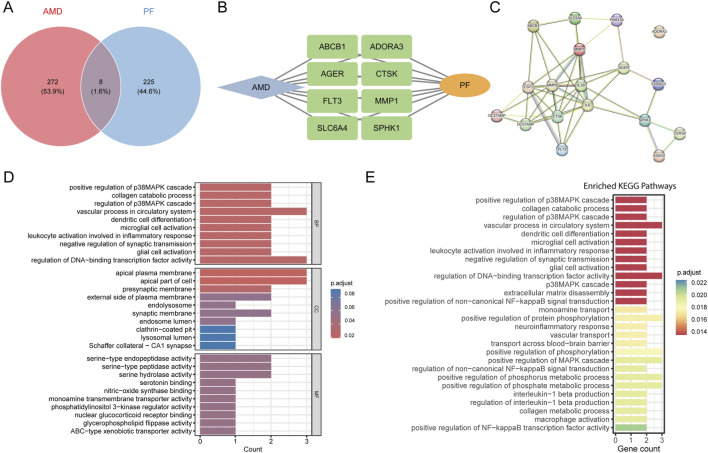
Identification of hub genes in AIPF and enrichment analysis. **(A)** Intersection of AMD target genes and PF target genes. **(B)** Drug-disease-gene network diagram. **(C)** PPI network diagram of target genes. **(D)** GO enrichment. **(E)** KEGG enrichment.

To clarify the pathogenic mechanisms of AIPF, we compiled gene sets associated with AMD exposure and PF from public databases and performed enrichment analysis. This analysis revealed that 280 genes were significantly associated with AMD, while 233 genes were significantly associated with PF. Notably, eight intersecting genes (MMP1, ADORA3, ABCB1, AGER, SLC6A4, CTSK, SPHK1, and FLT3) were shared by both AMD and PF, suggesting that AMD may induce PF through these hub genes. In the constructed PPI network, several key hub nodes with high connectivity were identified, including CTSK, AGER, FLT3, and MMP9. These molecules occupy central positions in the network topology, suggesting their potential involvement in critical biological processes. To identify key mechanisms underlying AIPF, we performed GO and KEGG analyses. As shown in Figure 4, several biological processes closely related to inflammatory responses, ECM remodeling, collagen metabolism, and the MAPK signaling pathway were significantly enriched.

Functional enrichment analysis of DGEs identified critical processes in AIPF. GO analysis highlighted significant enrichment in critical biological processes, including “positive regulation of p38MAPK cascade” “collagen catabolic process” “microglial cell activation” and “leukocyte activation involved in inflammatory response”. KEGG pathway analysis further established the central role of the “p38MAPK cascade” alongside “ECM disassembly” “positive regulation of NF-κB signaling” and “IL-1 production”. The coordinated enrichment of these pathways indicates a mechanistic framework where AMD triggers a pro-fibrotic response through p38 MAPK/NF-κB-driven inflammation and ECM remodeling.

### 4.4 Machine learning and SHAP analysis

**FIGURE 5:**
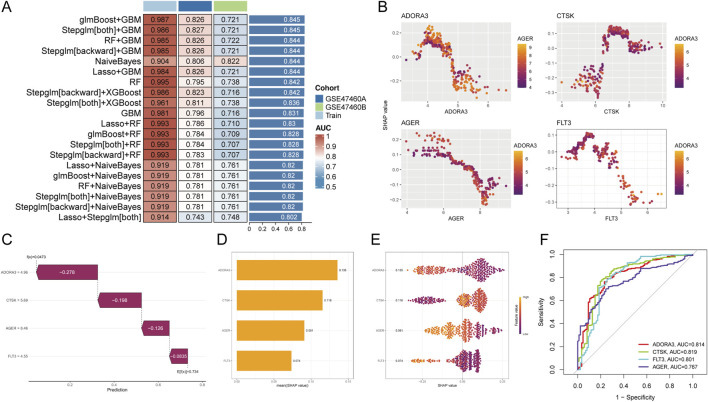
Identification and validation of hub genes in AIPF based on ML and SHAP analysis. **(A)** Heatmap compares the AUC of various ML models to determine the best predictive model (glmboost + GBM). **(B)** SHAP summary plot visualizes the contribution of genes to the GBD model. **(C)** An attempt to decompose the feature contributions of representative samples confirms the global importance observed in SHAP analysis. **(D)** SHAP analysis results for the intersecting genes, shown as a SHAP value bar chart. **(E)** Violin plot depicts the expression distribution of core genes in AIPF and the control group. The eigenvalues of CTSK are positively correlated with SHAP values, while ADORA3, AGER, and FLT3, show negative correlations. **(F)** ROC curves validate the clinical data for core genes.

We employed multiple ML algorithms to construct AIPF diagnostic and predictive models, evaluating their performance on independent cohorts. As shown in Figure 5A, all models were trained on our primary dataset and validated on cohorts GSE47460A and GSE47460B. Results show that the glmBoost + GBM model demonstrated outstanding and stable predictive performance, achieving the highest area under the curve (AUC) on the training set (AUC = 0.987) and maintaining robust accuracy in external validation (AUCs of 0.826 and 0.721, respectively). Through a two-stage integrated design, sparse feature selection based on generalized linear models boosting (glmBoost) is combined with robust nonlinear gradient boosting modeling (GBM), structurally achieving complementary dimension reduction and complex relationship modeling. This design effectively mitigates overfitting risks in high-dimensional data while leveraging GBM’s capability to capture complex interactions among key features, thereby enhancing the model’s generalization performance and predictive accuracy. The model’s rigor is doubly validated: through its robust performance maintained on an independent validation cohort, and through subsequent SHAP-based interpretability analysis that thoroughly elucidates its decision logic. Four hub genes were involved in glmBoost + GBM model construction: ADORA3, CTSK, AGER, and FLT3, The SHAP analysis scatter plot is shown in Figure 5B. SHAP interpretable analysis revealed distinct functional contributions: ADORA3 (SHAP value = 0.135), CTSK (SHAP value = 0.116), AGER (SHAP value = 0.091), FLT3 (SHAP value = 0.074) emerged as the most influential predictors. Among these, CTSK exhibited positive regulatory effects while ADORA3, AGER, FLT3 exhibited negative regulatory effects, in a representative sample, features were ranked according to their contribution (SHAP value). In a representative experiment, the analysis showed that ADORA3 (SHAP = −0.278) and CTSK (SHAP = −0.198) were the strongest positive drivers, significantly pushing the model’s predicted value from the baseline (E[f(x)] = 0.473) to a higher risk level (Figure 5C). Figures 5D,E shows the contribution (Figure 5D) and direction (Figure 5E) of the SHAP analysis. To further assess the viability of these core genes as diagnostic biomarkers, we constructed ROC curves. Figure 5F illustrates that all four hub genes exhibited strong diagnostic discrimination capability, with the area under the curve above 0.70. CTSK and ADORA3 exhibit the highest diagnostic efficacy, with AUC values of 0.819 and 0.814, respectively, followed by FLT3, and AGER indicating significant promise for clinical application.

### 4.5 Molecular docking and molecular dynamics simulation

**FIGURE 6:**
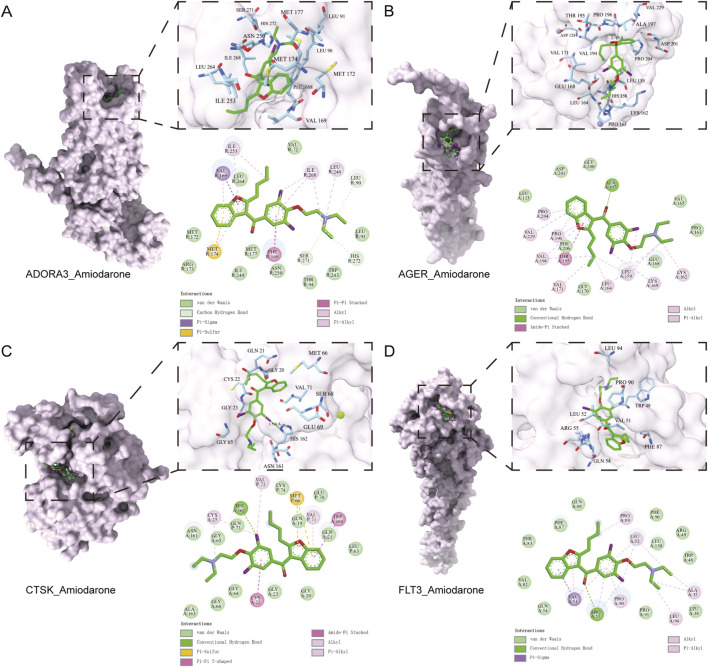
Molecular docking results. The molecular docking results of amiodarone with ADORA3 **(A)**, AGER **(B)**, CTSK **(C)**, and FLT3 **(D)** are presented.

Based on the SHAP analysis, the four most prominent potential target genes (CTSK, ADORA3, AGER, and FLT3) were prioritized for further investigation. To elucidate the molecular interactions between amiodarone and these targets, systematic molecular docking was conducted, followed by molecular dynamics simulations. The docking results demonstrated that AMD exhibited strong spontaneous binding affinity to all tested proteins (Vina Score ≤ −6.0 kcal/mol), with the strongest binding observed for AGER (−7.6 kcal/mol) and ADORA3 (−7.5 kcal/mol). Detailed interaction analysis revealed that amiodarone stabilizes within the active pockets of each protein through multiple non-covalent interactions, including hydrogen bonds, extensive hydrophobic contacts, π-π stacking, and van der Waals forces, providing preliminary insight into its potential multi-target mechanism. The results of molecular docking are shown in Table 3 and Figure 6.

**TABLE 3:** Molecular docking results.

Compound	Protein	PDB ID	Vina score (kcal/mol)
Amiodarone	ADORA3	9EBI	−7.5
	AGER	3CJJ	−7.6
	CTSK	7QBM	−7.3
	FLT3	7ZV9	−6.9

**FIGURE 7:**
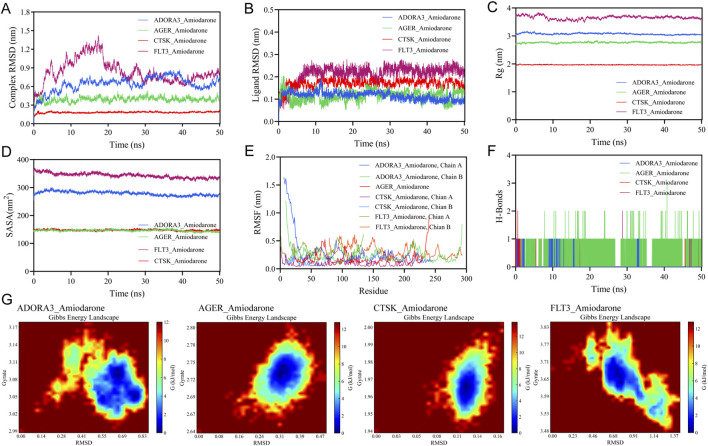
50 ns molecular dynamics simulation analysis of the complex. **(A)** RMSD curve of the complex, **(B)** RMSD curve of the small molecule, **(C)** Rg curve of the protein, **(D)** SASA curve of the protein, **(E)** RMSF curve of the protein, **(F)** Hydrogen bond evolution curve of the complex, **(G)** Gibbs FEL of the AMD-complex.

To further investigate the dynamic binding characteristics of the complexes, we performed 50 ns molecular dynamics simulations and binding free energy calculations. Analyses of RMSD (Brüschweiler, 2003; He et al., 2023), RMSF (Baskin, 1968), Rg (Davoudmanesh and Mosaabadi, 2018), and SASA (Richmond, 1984; Castrosanto et al., 2023) consistently indicated that the amiodarone-protein complexes maintained good structural stability throughout the simulation, with the CTSK-amiodarone complex displaying the highest conformational rigidity. Free energy landscape (FEL) analysis thermodynamically confirmed that the CTSK system possessed the most concentrated low-energy conformational basin. More importantly, MM/PBSA calculations quantitatively verified that the binding of amiodarone to ADORA3, CTSK, and FLT3 was highly thermodynamically favorable (ΔG_bind_ = −47.61, −39.45, and −36.01 kcal/mol, respectively), with Van Der Waals interactions identified as the major energetic contributor. Together, these results provide computational evidence at both the atomic dynamical level and the thermodynamic quantitative level, supporting the stable and preferential binding of AMD to the aforementioned target proteins and offering a solid foundation for its potential multi-target pharmacological effects. Analysis of Gibbs FEL maps indicates that all four protein-ligand complex systems exhibit highly stable binding conformations. The free energy distributions for each system reveal a single, continuous, and narrowly concentrated minimum energy region. The deep blue energy traps are broad and smooth, suggesting minimal conformational fluctuations and high structural rigidity in the bound complexes, with no apparent metastable states or conformational transition pathways. Therefore, AMD forms strong and stable specific interactions with CTSK, ADORA3, AGER, and FLT3, providing crucial evidence of conformational stability for subsequent drug design or mechanism studies. The results of molecular dynamics simulations are shown in Table 4 and Figure 7.

**TABLE 4:** Energy decomposition analysis by MM/PBSA.

Contribution	ΔE_vdW_	ΔE_elec_	ΔG_polar_	ΔG_nonpolar_	ΔG_gas_	ΔG_solv_	ΔG_bind_
ADORA3_Amiodarone	−53.12	−2.20	14.47	−6.77	−55.32	7.71	−47.61
AGER_Amiodarone	−28.84	−5.30	15.13	−3.68	−34.14	11.45	−22.69
CTSK_Amiodarone	−51.99	−6.08	21.80	−6.18	−58.07	15.63	−42.45
FLT3_Amiodarone	−46.00	−7.66	18.29	−5.81	−53.66	12.48	−41.18

### 4.6 *In vitro* experimental validation

**FIGURE 8:**
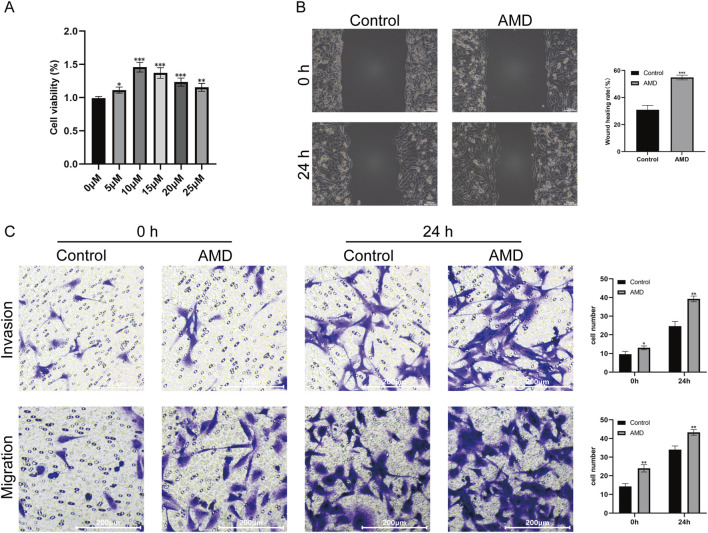
Effects of AMD on BEAS-2B cell migration, invasion capacity, and hub gene expression. **(A)** CCK8 assay determines the optimal concentration for AMD intervention in BEAS-2B cell. **(B)** The wound healing assay demonstrated significantly accelerated wound closure after 24 h of AMD treatment (scale bar: 100 μm). **(C)** Transwell invasion/migration assay revealed significantly increased BEAS-2B cell density after 24-h AMD treatment (scale bar: 200 μm) (*p < 0.05, **p < 0.01).

We used a CCK-8 assay to assess AMD’s impact on BEAS-2B cell viability and find the best intervention concentration. In Figure 8A, cell viability was not significantly affected by AMD concentrations from 1 μM to 10 μM. Cell viability peaked at 10 μM AMD concentration. In the 10–25 μM range, cell viability significantly decreased. In following trials, 10 μM AMD was found to be the most efficient intervention concentration for modulating BEAS-2B cells without causing severe cytotoxicity. The wound healing assay demonstrated a similar pro-migratory effect induced by AMD. While wound closure rates were comparable at the initial stages, the AMD-treated group displayed a significantly accelerated wound closure over 24 h. The relative wound healing area was substantially smaller in the AMD group compared to the control, further confirming that AMD stimulation enhances the lateral migration of BEAS-2B cells in a two-dimensional model (Figure 8B). To functionally assess the impact of AMD on the motility and invasive potential of BEAS-2B cells, we performed transwell migration and invasion assays alongside a wound healing assay. At the 0-h time point, no discernible difference in cell migration or invasion was observed between the control and AMD-treated groups. However, after a 24-h incubation period, AMD-treated cells exhibited a significantly enhanced migratory and invasive capacity compared to the control. The significant increase in the number of cells migrating through Matrigel-coated membranes suggests that AMD exposure enhances cell motility and confers a pronounced pro-invasive phenotype (Figure 8C). These functional assays demonstrate that AMD intervention significantly enhances the migratory, invasive, and wound healing capabilities of pulmonary epithelial cells, thereby revealing the toxicological mechanisms underlying AIPF.

**FIGURE 9:**
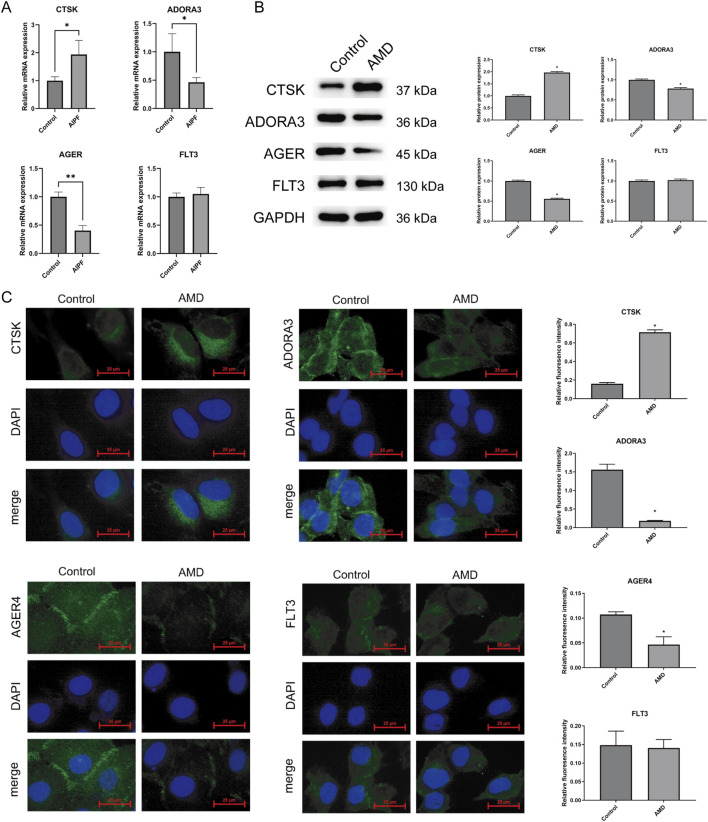
mRNA and protein expression of AIPF hub genes. **(A)** qRT-PCR analysis revealed upregulation of CTSK mRNA expression and downregulation of ADORA3 and AGER mRNA expression following AMD treatment. **(B)** Western blot was used to detect the expression of target proteins in the Control group and AMD group. **(C)** Immunofluorescence validated key protein expression and localization: CTSK (green) showed enhanced granular expression in the cytoplasm; ADORA3 (green) exhibited reduced ring-like expression at the cell membrane; AGER (green) decreased in both membrane and cytoplasm; FLT3 (green) showed no significant change; nuclei were labeled with DAPI (blue) (scale bar: 50 μm) (*p < 0.05, **p < 0.01).

Furthermore, we analyzed the mRNA expression levels of four hub genes (CTSK, ADORA3, AGER, and FLT3) in AMD-induced BEAS-2B cells using qRT-PCR technology. Quantitative analysis indicated that, in comparison to the control group, CTSK mRNA expression shown significant upregulation (*p* < 0.05), whereas both ADORA3 and AGER displayed substantial downregulation (*p* < 0.05, *p* < 0.01). Nevertheless, under identical treatment settings, FLT3 expression levels exhibited no statistically significant alterations. The results indicate that the overexpression of CTSK and the downregulation of ADORA3 and AGER may be pivotal in the pulmonary epithelial cell response to AMD and the probable advancement of PF (Figure 9A). Western blot analysis revealed differential expression profiles of target proteins between control group and AMD group.​ Compared to the control group, the expression of CTSK was markedly upregulated in the AMD group (*p* < 0.05). In contrast, the level of ADORA3 and AGER showed a distinct downregulation (*p* < 0.05, *p* < 0.05). The expression of FLT3 appeared comparable between the two groups, indicating no substantial change (Figure 9B). Immunofluorescence analysis further validated the protein-level alterations of these hub genes and delineated their distinct subcellular localizations in BEAS-2B cells. Consistent with the mRNA data, CTSK protein expression was significantly upregulated and exhibited a characteristic granular pattern co-localized with lysosomal markers within the cytoplasm. Conversely, ADORA3 displayed a marked downregulation and was predominantly localized to the plasma membrane, exhibiting a perimenbranous ring-like staining. AGER protein expression was also substantially reduced, showing a combined localization pattern with signal present both at the plasma membrane and diffusely throughout the cytoplasm. In contrast, FLT3 protein levels showed no significant change following AMD treatment, and it maintained its expected specific localization to the cell membrane (Figure 9C). These results collectively confirm the differential expression of CTSK, ADORA3, and AGER at the protein level and define their precise cellular compartments, providing crucial morphological evidence for their potential roles in AMD-induced epithelial cell injury and the subsequent pathogenesis of pulmonary fibrosis.

In summary, these data demonstrate that AMD exposure reprograms pulmonary epithelial cells through upregulation of CTSK and downregulation of ADORA3 and AGER, leading to an enhanced migratory and invasive phenotype, highlighting a pivotal role for CTSK-driven epithelial dysfunction in the progression of PF.

## 5 Discussion

AMD tends to accumulate in alveolar type II epithelial cells, disrupting phospholipid metabolism and provoking oxidative stress, inflammation, and immune responses, which activate fibrosis signaling pathways such as TGF-β1. This process induces EMT and EndMT in alveolar cells, stimulates fibroblast proliferation, and promotes extensive collagen synthesis, ultimately resulting in PF (Dong et al., 2017; Li et al., 2022). Despite considerable advancements in clarifying the pathological mechanism of AIPF, the exact identification of its upstream initiating factors and the core genes of AIPF remains unresolved, leading to a limited comprehension of the intricate cascade reaction mechanism that instigates the disease. Through an integrative approach combining ML algorithms and SHAP analysis, we conducted a stepwise screening process to identify core genes in AIPF, ultimately pinpointing CTSK, ADORA3, and AGER as the most robust predictors and potential key mediators.

CTSK (Cathepsin K) is a lysosomal cysteine protease with potent collagen degradation activity and is a member of the C1 family. It is most abundantly expressed in bone-resorbing cells (osteoclasts), but its protein and activity have also been detected in lung tissue fibroblasts, bronchial epithelial cells, and lung macrophages (Díaz et al., 2000; Meng et al., 2022). CTSK can achieve rapid clearance of the ECM by directly cleaving type I and type III collagen fibers and elastin, a function that is particularly critical in maintaining lung matrix homeostasis (Srivastava et al., 2008). However, In the pathological process of PF, the role of CTSK shows bidirectional regulation. On the one hand, CTSK plays a profibrotic role. Recent studies have shown that CTSK acts as a key regulator of fibroblast activation, driving fibrosis by remodeling glutamine metabolism and upregulating collagen synthesis genes. A recent study demonstrates that CTSK promotes PF by driving fibroblast activation and collagen synthesis via a SNX9-mediated endocytotic pathway, which facilitates TGF-β1/SMAD3-dependent glutaminase upregulation and subsequent glutamine metabolic reprogramming (Chen M. et al., 2025). On the other hand, CTSK can inhibit fibrosis. Experimental data show that CTSK overexpression enhances collagen degradation in the lungs, reducing bleomycin-induced collagen deposition and pulmonary resistance, thereby counteracting fibrosis. This protective effect is closely related to its specific collagen degradation activity within the phagosome of lung macrophages and is negatively regulated by TGF-β1 (Fabrik et al., 2023). Kristy et al. demonstrated that IGF-II drives PF in systemic sclerosis by activating a profibrotic program through the transcription factor SOX9, which concurrently upregulates collagen synthesis/modifying enzymes and represses collagen-degrading cathepsins, including CTSK, which suggests that CTSK plays a potential anti-fibrotic role in PF, and its expression is inhibited by the pro-fibrotic core signal IGF-II (Waldrep et al., 2023). Given the paradoxical role of CTSK in pulmonary fibrosis, the observation that it is upregulated in our AMD-induced BEAS-2B cell model suggests a context where its pro-fibrotic effects are dominant.

ADORA3 (Adenosine A_3_ receptor) is a member of the G protein-coupled receptor (Gi/Go) family (Borea et al., 2015). It regulates inflammation, apoptosis, and fibrosis by inhibiting adenylate cyclase, reducing intracellular cAMP levels, and activating signaling pathways such as MAPK and PI3K-Akt (Morschl et al., 2008). The role of ADORA3 in PF is also bidirectional. On the one hand, ADORA3 may negatively impact the advancement of PF by diminishing the secretion of proinflammatory cytokines (including TNF-α and IL-1β), modulating the conversion of fibroblasts into myofibroblasts, and affecting the synthesis of type I and III collagen along with the activity of matrix metalloproteinases (MMPs) (Kreckler et al., 2006; Martin et al., 2006; Young et al., 2006). AIPF is chiefly linked to drug accumulation in alveolar epithelial cells and macrophages, lysosomal dysfunction, and autophagy dysregulation (Chang et al., 2007). Impaired lysosomal activity disrupts endocytosis, degradation, and recycling of membrane receptors, potentially modifying ADORA3 cell surface expression and signaling. Research indicates that AMD enhances lysosomal biogenesis through the activation of transcription factor EB (TFEB), while simultaneously causing disruptions in the autophagy-lysosomal pathway, which may elucidate the imbalance in ADORA3 signaling(Chang et al., 2007). In this environment, the anti-inflammatory and anti-fibrotic roles of ADORA3 may be compromised, resulting in lung inflammatory cell infiltration, heightened TGF-β activation, and faster collagen accumulation. Therefore, AMD-induced lysosomal dysfunction perturbs ADORA3-mediated anti-inflammatory and anti-fibrotic signaling, thereby exacerbating AIPF. On the other hand, A review by Della Latta et al. highlights the profibrotic role of ADORA3, noting that its expression is elevated in chronic lung diseases and that its signaling contributes to pulmonary inflammation and airway remodeling, key processes in fibrosis (Della Latta et al., 2013). In some studies, ADORA3 has been identified as a risk gene associated with poor prognosis in IPF, but the specific molecular mechanisms of ADORA3 have not been elucidated in detail (He et al., 2024). Therefore, the reduced expression of ADORA3 in AMD-induced BEAS-2B cells in this study, consistent with impaired anti-inflammatory and anti-fibrotic signaling pathways mediated by this receptor, may have weakened the protective role of alveolar epithelial cells against drug-induced injury.

AGER (Advanced glycosylation end product-specific receptor) also plays a dual regulatory role in AIPF. On the one hand, AGER, highly expressed in lung tissue, can bind to ligands such as HMGB1 and S100 proteins, activating the NF-κB, MAPK, and PI3K/Akt pathways, thereby upregulating TGF-β1 and CTGF expression and promoting epithelial-mesenchymal transition, fibroblast proliferation, and collagen deposition (Mahavadi et al., 2015; Budin et al., 2022). AMD, through accumulation in alveolar type II cells, inhibits lysosomal phospholipase activity and induces apoptosis and autophagy-dependent cell death, further exacerbating fibrosis signaling (Mahavadi et al., 2015; Harb et al., 2023). AGER which binding diverse damage-associated molecular patterns (DAMPs) such as HMGB1 and S100s, propagates a persistent pro-inflammatory response via NF-κB activation, establishing a pathogenic positive feedback loop(Dong et al., 2022; Twarda-Clapa et al., 2022; Chen R. et al., 2025). This signaling axis drives chronic inflammation and aberrant tissue remodeling, key processes in the pathogenesis of PF, thereby positioning AGER inhibition as a promising therapeutic strategy for this condition (Hudson and Lippman, 2018; Chen R. et al., 2025). On the other hand, soluble AGER (sAGER) and its truncated isoenzymes act as decoy receptors, competitively binding to ligands to weaken full-length RAGE-mediated signaling, thereby inhibiting the release of inflammatory cytokines and collagen gene expression, exerting an anti-fibrotic protective effect (Yamaguchi et al., 2017). In asbestos, silica, or aging models, the absence of RAGE exacerbates fibrosis or leads to spontaneous fibrosis-like changes. The mechanism is complex, and its effects encompass pathogenic processes such as lung injury response, type II immune response, and cellular senescence, indicating that AGER has a physiological protective role in maintaining the integrity of the lung epithelium (Ramsgaard et al., 2010; Perkins and Oury, 2021). Similarly, studies by Markus et al. indicate that AGER exerts a protective effect in the lungs, and its downregulation in IPF promotes a pro-fibrotic cellular phenotype, including enhanced proliferation and migration capabilities of fibroblasts and epithelial cells (Queisser et al., 2008). In light of its dual function in AIPF, the observed downregulation of AGER in our AMD-induced BEAS-2B model indicates a context-specific, protective adaptation that mitigates pro-fibrotic signaling.

FLT3 (FMS-like tyrosine kinase 3) is a receptor tyrosine kinase highly expressed in hematopoietic stem/progenitor cells and early myeloid precursors (Koga et al., 2025). Activation of its ligand, FLT3-L, can promote cell proliferation and survival through signaling pathways such as PI3K/AKT, RAS/RAF/MEK/ERK, and STAT5. Although the primary mechanism of AIPF has been shown to involve TGF-β1-mediated epithelial-mesenchymal transition, phospholipid deposition, and inflammatory cell infiltration, current literature has not shown a direct role for FLT3 in this pathological process, suggesting that its function in AIPF may be limited to indirect regulation of inflammatory cell recruitment and activation. Further experiments are needed to verify its potential mechanism of involvement. Therefore, this study confirms that FLT3 expression shows no significant change in AMD-induced BEAS-2B alveolar epithelial cells, suggesting that it may not play a direct role in the cytotoxic response of AIPF lung epithelial cells.

Although genes such as MMP1, ABCB1, SLC6A4, and SPHK1 exhibit low SHAP values, they may still contribute to the pathogenesis of PF. This assertion is supported by multiple studies emphasizing the multifaceted roles these genes play in disease mechanisms, even when their individual contributions are minimal in certain computational models. MMP1 (Matrix Metalloproteinase-1) exhibits a paradoxical, context-dependent function in PF (Lei et al., 2025). During the acute injury repair phase, elevated MMP1 expression is considered a mechanism contributing to self-protection and tissue remodeling (Zheng et al., 2023), however, during the sustained progression phase of fibrosis, persistently high MMP1 expression may act as a driver of fibrosis or tumorigenesis(Ishida et al., 2023). Notably, MMP-1 facilitates the detachment of epithelial and endothelial cells from monolayers and promotes their migration, a key step in initiating EMT and EndMT, thereby contributing to fibrotic progression (Du et al., 2024; Khalili-Tanha et al., 2025). The role of ABCB1 (ATP-binding cassette subfamily B member 1), also known as multidrug resistance protein 1 (MDR1), represents an emerging area of investigation that intersects drug resistance, cellular detoxification, and fibrotic signaling pathways (Szöllősi et al., 2018; D'Agnano et al., 2024). Although ABCB1 is not conventionally regarded as a central mediator of pulmonary fibrosis, accumulating evidence suggests a potential role in regulating disease-relevant processes, including toxin clearance, DNA damage response, and possibly EMT and fibroblast activation. SLC6A4 (Serotonin transporter gene) may be involved in the pathogenesis of pulmonary fibrosis by regulating the transport and homeostasis of serotonin (5-HT), and its mechanism may involve effects on fibroblast activation and the polarization of immune cells such as macrophages. Dysregulation of SLC6A4 function may contribute to the progression of fibrotic processes, whereas targeted modulation of its activity holds potential therapeutic implications for pulmonary fibrosis (Dai et al., 2022). SPHK1 (Sphingosine Kinase 1) primarily promotes fibroblast proliferation, differentiation into myofibroblasts, and excessive collagen production by upregulating S1P levels, activating the Hippo/YAP pathway and mitochondrial ROS, ultimately leading to hardening of lung tissue (Huang et al., 2020). S1P concurrently regulates cytoskeletal remodeling (mostly via S1PR2 and S1PR3) (Fohmann et al., 2023) and undermines monolayer stability (facilitated by S1PR1) (Garnier and Vilgrain, 2023), together fostering tissue stiffening and fibrotic advancement in the lung. Therefore, despite lower SHAP values in specific ML models, MMP1, ABCB1, SLC6A4, and SPHK1 may synergistically contribute to the development and progression of PF through key biological processes such as matrix remodeling, cellular defense, and signal transduction.

In summary, the pathogenesis of AIPF is caused by the dysregulation of core genes, with the dual roles of CTSK and AGER being particularly prominent. CTSK bidirectionally regulates ECM dynamics: it directly degrades collagen and activates fibroblasts to promote fibrosis. Simultaneously, the AGER pathway maintains a dynamic balance: membrane-bound AGER drives pro-fibrotic pathways (NF-κB/MAPK/PI3K-AKT), while its soluble homolog (sAGER) acts as a decoy receptor to exert anti-fibrotic effects. Crucially, the protective anti-fibrotic signaling mediated by the ADORA3/cAMP axis is impaired by AMD-induced lysosomal dysfunction. Although MMP1, ABCB1, SLC6A4, and SPHK1 may play roles in specific pathological pathways of pulmonary fibrosis, their SHAP values in the current ML model are relatively low. This indicates limited evidence supporting their roles as independent key drivers or core biomarkers. Therefore, the pathogenesis of AIPF stems from the disruption of the relationship between these pro-fibrotic factors and impaired protective mechanisms. In addition, the imbalance between profibrotic drivers and compromised antifibrotic defenses may extend beyond parenchymal fibrosis to critically influence pulmonary vascular dysfunction—warranting focused investigation into the pathogenesis of pulmonary hypertension in fibrotic lung disease, which may become a hot topic for future research (Weinstein et al., 2024).

## 6 Conclusion

We integrated transcriptome data with *in vitro* cell experiments to identify three core genes involved in AIPF (CTSK, ADORA3, and AGER). Currently, the roles of CTSK, ADORA3, and AGER in PF remain inconclusive. Whether their ultimate effect promotes or inhibits fibrosis likely depends on the etiology, stage of development, and overall signaling environment within the organism. Through ML and SHAP interpretability analyses, we determined the predictive value of these genes and validated the interaction between AMD and these targets using molecular docking. *In vitro* experiments verified their expression levels. This study provides new insights into the pathogenesis of AIPF and identifies key genes worthy of further investigation.

## Data Availability

The data supporting the findings of this study are available from the corresponding author, Rui Fan (fanruialbert@foxmail.com), upon reasonable request. The transcriptomic data analyzed during this study are publicly available in the Gene Expression Omnibus (GEO) repository under accession numbers GSE17978, GSE24206, GSE32537, GSE40839, and GSE47460.

## Glossary

ABC: Area Under the Curve
ABCB1: ATP Binding Cassette Subfamily B Member 1
ADORA3: Adenosine A3 Receptor
AGER: Advanced Glycosylation End-Product Specific Receptor
AIPF: Amiodarone-Induced Pulmonary Fibrosis
AIPT: Amiodarone-Induced Pulmonary Toxicity
AMD: Amiodarone
cAMP: Cyclic Adenosine Monophosphate
CTSK: Cathepsin K
DAMPs: Damage-Associated Molecular Patterns
DEGs: Differentially Expressed Genes
ECL: Enhanced Chemiluminescence
ECM: Extracellular Matrix
EMT: Epithelial-Mesenchymal Transition
EndMT: Endothelial-Mesenchymal Transition
FEL: Free Energy Landscape
FLT3: FMS-Like Tyrosine Kinase 3
GBM: Gradient Boosting Machine
GO: Gene Ontology
KEGG: Kyoto Encyclopedia of Genes and Genomes
MAPK: Mitogen-Activated Protein Kinase
ML: Machine Learning
MMP1: Matrix Metalloproteinase-1
PCA: Principal Component Analysis
PDB: Protein Data Bank
PF: Pulmonary Fibrosis
PPI: Protein-Protein Interaction
qRT-PCR: Quantitative Real-Time Polymerase Chain Reaction
RAGE: Receptor for Advanced Glycation End-Products
ROC: Receiver Operating Characteristic
SHAP: Shapley Additive exPlanations
SLC6A4: Solute Carrier Family 6 Member 4
SMILES: Simplified Molecular-Input Line-Entry System
SPHK1: Sphingosine Kinase 1
TFEB: Transcription Factor EB
TGF-β1: Transforming Growth Factor Beta 1
WGCNA: Weighted Gene Co-Expression Network Analysis
